# *In memoriam* Professor Jean-Antoine Rioux (1925-2017)

**DOI:** 10.1051/parasite/2018012

**Published:** 2018-03-13

**Authors:** René Houin, Nicole Léger, Jean Dupouy-Camet, Patrick Bastien, Gérard Luffau

**Affiliations:** 1 Académie Vétérinaire de France, 34 rue Bréguet, 75011 Paris France; 2 UFR de Pharmacie, Université de Reims Champagne-Ardenne, 51096 Reims France; 3 Faculté de Médecine Paris Descartes, Paris France; 4 Département de Parasitologie-Mycologie, CHU de Montpellier/Faculté de Médecine, UMR “MiVEGEC” (CNRS 5290/IRD 224 /UM), Université de Montpellier, Av. Charles Flahault, Montpellier Cedex 5 France; 5 Unité de virologie et immunologie moléculaires, Inra, Jouy-en-Josas France

Born in Naucelle (Aveyron, south-central France), in a family firmly rooted in the Limousin region, Jean-Antoine Rioux (Figure 1) spent his childhood in Le Vigan, in the heart of the wild Cevennes mountain range. It is undoubtedly there that he acquired his passion for studying nature, which underpinned his activities during his whole life. This fascination led him to deepen the study of botany, a skill which gave him the opportunity later on, between 1977 and 1993, to become the director of the Botanical Garden (“*Jardin des Plantes*”) of the city of Montpellier [12]. He devoted many years to this task with talent and perseverance, preserving and enriching the plant collections, renovating the greenhouses, promoting the garden’s image, and ensuring its protection through its classification among “Noticeable Sites and Landscapes” in 1982 and “Historical Monuments” in 1992. To this initial passion was added a strong medical vocation. He was a brilliant medical student during his studies in Montpellier, and graduated top of the class in the “*Concours de l’Internat*” (a competitive examination to recruit hospital residents) in 1951. He was then appointed as Assistant Professor in Montpellier University Hospital and specialized in dermatology and lung diseases. But in 1952, he soon found the area in which he reached his greatest achievements, by joining the Laboratory of Medical Natural History headed by Hervé Harant, skillful naturalist and parasitologist with great scientific understanding. Jean-Antoine Rioux then specialized in parasitology, and he continued his whole career in this organization termed the “Laboratory of Parasitology” which later became the “Laboratory of Medical Ecology and Parasite Pathology”. Jean-Antoine Rioux built a remarkable establishment, recognized and supported by research authorities, which allowed him to conduct research work leading to more than 500 publications.

**Figure 1:**
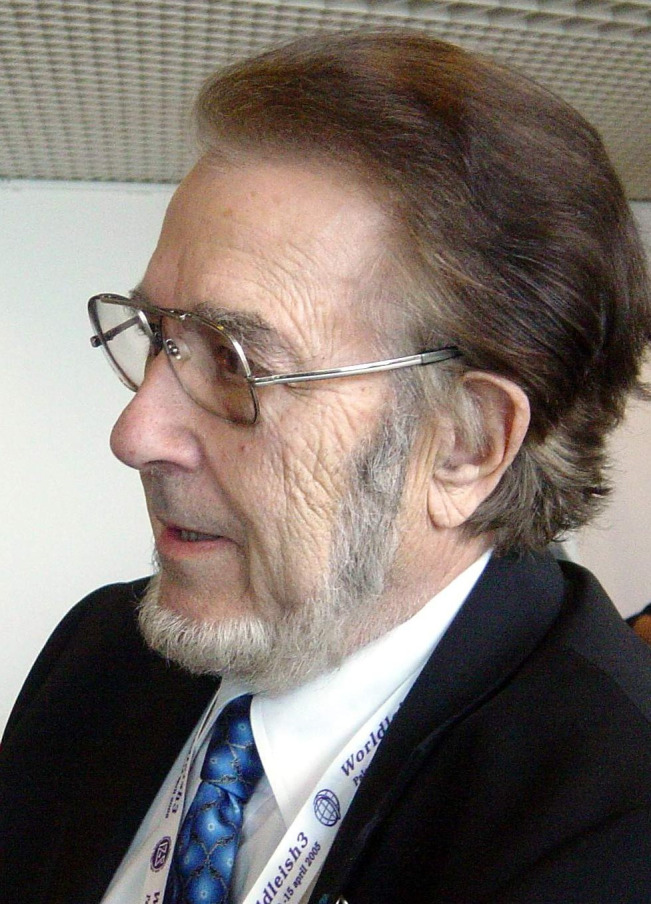
Professor Jean-Antoine Rioux (1925-2017).

## Early work on malaria and mosquitoes

In French faculties of medicine, teaching the epidemiology of malaria has always been one of the major goals of the parasitology curriculum. The young Jean-Antoine Rioux aimed at transforming this teaching topic into a research activity. During the preparation of his PhD on the “*Culicidae du Midi Méditerranéen*”, he had acquired strong entomological training, which he used to study malaria in Languedoc-Roussillon, proposing the concept of “unstable malaria”. This concept would be taken up later, on the occasion of several epidemiological missions in North Chad (Borkou-Ennedi-Tibesti). The persistence, even temporary, of malaria parasites in oasis inhabitants especially in sedentary children, was indicative of *in situ* transmission and of the real presence of vectors. In fact, he found larvae of *Anopheles gambiae* complex in the overflow sites of the Faya-Largeau region and one year later, the adults were captured in an oasis dwelling. During these surveys, 25 Culicidae species were identified, providing important bio-geographical clarifications. In the Mediterranean South of France, he also studied variable autogenesis of halophilic sublittoral *Aedes (Ochlerotatus) detritus*. This variability suggested either simple genetic polymorphism or a true segregating population (cryptic species). Analysis of allelic isozyme frequencies in adults from the same larval breeding habitat (in collaboration with Nicole Pasteur) confirmed the second hypothesis. This twin species was described under the binomial appellation *Aedes (Ochlerotatus) coluzzii* Rioux, Guilvard & Pasteur [10]. *A. coluzzii* could be bred without any difficulty in an insectarium, whereas to obtain fertile progeny with *A. detritus s. st*., artificial fertilization was necessary. Jean-Antoine Rioux’s knowledge of entomology led him to perform a major ecological survey on *Leptoconops irritans*, a particularly aggressive Ceratopogonidae species, found in coastal areas from Camargue to Roussillon [3]. The objective of this action was to identify larval biotopes and to specify seasonal dynamics. A sampling transect was carried out for two years in Middle Camargue. The results clarified the situation and functioning of emergence sites of *L. irritans*: the most productive phytocenosis was formed by the perennial Chenopodiaceae groups, forming the essential parts of halophilic ecosystems of Middle and Lower Camargue. It was also on this basis that integrated pest management against “nuisance mosquitoes” was proposed (in French we use the word *démoustication* invented by Hervé Harant). The concept of nuisance concerns hematophagous species responsible only for discomfort and not for the transmission of pathogens (vectors). In order to implement measures to limit these species, it was necessary to obtain ecological data on all stages of insect development. This situation is quite different from that of vector-borne diseases, where the vector is only the entomological component of a pathogenic complex including vertebrate hosts (so-called reservoirs), humans and the pathogen itself. It is then necessary to act on compartmentalized cyclical systems; the control of which implicates very different operational strategies (integrated control). These strategies have later been developed for controlling leishmaniasis and will be detailed below. As mentioned above, mosquito control in Languedoc-Roussillon concerned four highly harmful species: *Culex pipiens*, *A. caspius*, *A. detritus* and *A. coluzzii*. The initiative, launched by the “*Entente Interdépartementale pour la Démoustication du Languedoc-Roussillon*” (EID Languedoc-Roussillon) created in 1958, was led by the interdepartmental tourism development authorities of the Languedoc-Roussillon region (“Mission Racine”) created in 1963. In sublittoral wetlands, suffering from a high mosquito nuisance, priority was given to anti-larval control and the fight against adult stages was only used in the event of failure of the latter, or to ensure the protection of tourist resorts from the clouds of adults coming from neighboring untreated sites (e.g., natural reserves). This was the case of La Grande Motte and Port-Camargue resorts, regularly invaded by mosquitoes from Camargue. In a few years, this strategy significantly reduced this problem with a minimum number of negative effects on the surrounding flora and fauna. At the same time, the fight against the domestic species *C. pipiens* was based on the screening and mapping of urban and peri-urban larval biotopes (cellars, sewers, septic tanks, ornamental ponds, rainwater receptacles, containers, etc.). In most cases, filling of larval habitats (e.g. sanitary voids), their cover by polystyrene beads (e.g. temporally flooded cellars) or even their definitive removal (e.g. abandoned containers) were sufficient to control the problem. Insecticides were used only as a last resort because of resistance phenomena. In addition, education and public awareness played a major role in this control program. Conceived more than half a century ago, the “*Entente Interdépartementale pour la Démoustication du Languedoc-Roussillon*” has remained remarkably effective and is an excellent example of successful integration between politicians, scientists and operators [14]. It resulted from the competence of the teams gathered by Jean-Antoine Rioux and his successors. This structure became a leading scientific center, both for French and foreign students and, also for experienced researchers. Moreover, after its success in the 1960s, the method was implemented in other regions of France (e.g. EID Atlantique, Rhône-Alpes, Alsace). In Guadeloupe, still in collaboration with the EID, a phyto-ecological map of the mangrovial larval habitats was drawn at a 1:25000 scale. Several countries later asked for assistance from the Montpellier group, including Tunisia, Morocco, Spain, Greece, Cyprus, and Canada.

**Figure 2:**
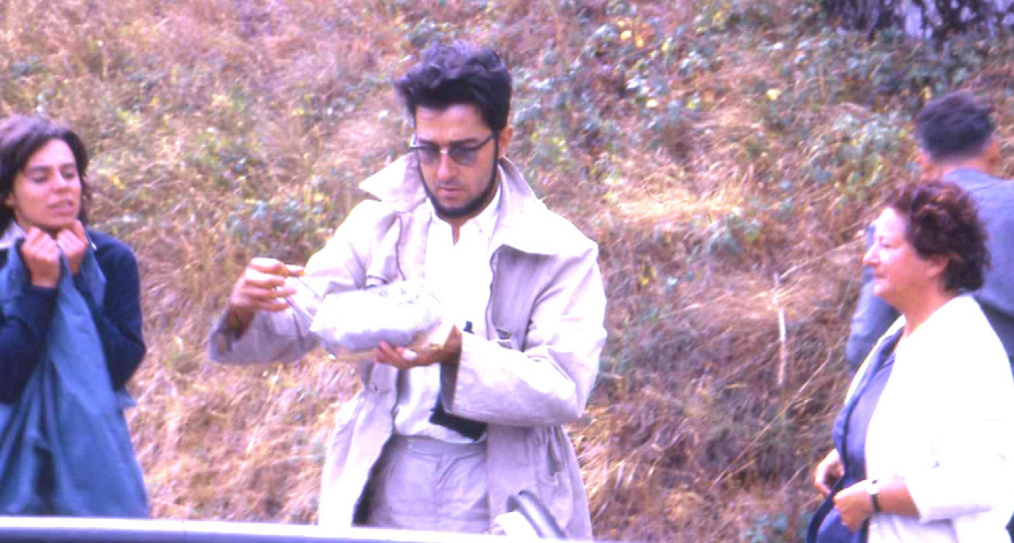
Jean-Antoine Rioux sampling in the field with Yvonne Campana-Rouget (right) & Françoise Deltour.

## Plague and schistosomiasis

Before discussing his contribution to the study of leishmaniasis and introducing the corresponding studies on pathogenic complexes, let us mention two programs, not targeting vectors but rather reservoir hosts of the plague and schistosomiasis. These programs were relatively limited in duration, but produced highly significant results. The first, a project carried out in his early career, was conducted with Yves-Jean Golvan and the Pasteur Institute of Teheran, Iran in 1960 [2]. It dealt with the Gerbillidae reservoirs of the enzootic plague in Iranian Kurdistan *(Meriones* spp.). This study, conducted on an annual biological cycle, identified the biotopes of these major rodent reservoir species (spatial localization of populations, geomorphological characteristics of their burrows, specific trophic preferences, phyto-ecological mapping of risk areas), and defined the roles of susceptible species (*Meriones persicus*) and resistant species (*M. vinogradovi*) between which the bacillus circulates. At the end of the survey, traditional agro-pastoral practices (extensive pastures, “dry” grain farming) were considered to be major determinants of plague foci. The second study began in 1972 under the authority of the *Délégation Générale* *à la Recherche Scientifique et Technique* (DGRST). Jean-Antoine Rioux had the operational responsibility of a project focused on the integrated control of intestinal schistosomiasis in Guadeloupe, French Caribbean. This program involved multiple teams of specialists: ecologists, epidemiologists, parasitologists, malacologists, water biologists, sociologists, and physicians [6]. The watershed, with its hydrographic network, its vector snails and its human occupation, constituted the unifying element (epidemiology of the landscape). After ten years of research *in natura*, accompanied by integrated interventions (management of contaminated watercourses, detection and treatment of clinical cases and healthy carriers, sanitation, and health education), anthroponotic transmission was successfully interrupted. However, some sylvatic micro foci (e.g. mangroves), involving black rats as reservoirs, remained active (zoonotic transmission). As a result, continued surveillance was recommended. Despite their apparent diversity, all these research themes are underpinned by the concepts and methods of general ecology applied to classical epidemiology. The work carried out has led to the individualization of a new discipline: eco-epidemiology, now recognized by several organizations in France, such as the CNRS, and international bodies including the WHO, FAO, and OIE. Most of his research studied only one of the members of the “pathogenic complex”, either the pathogenic agents themselves or their vectors or reservoirs. Other works have dealt with more finalized research, such as the “integrated control” of nuisance mosquitoes.

**Figure 3:**
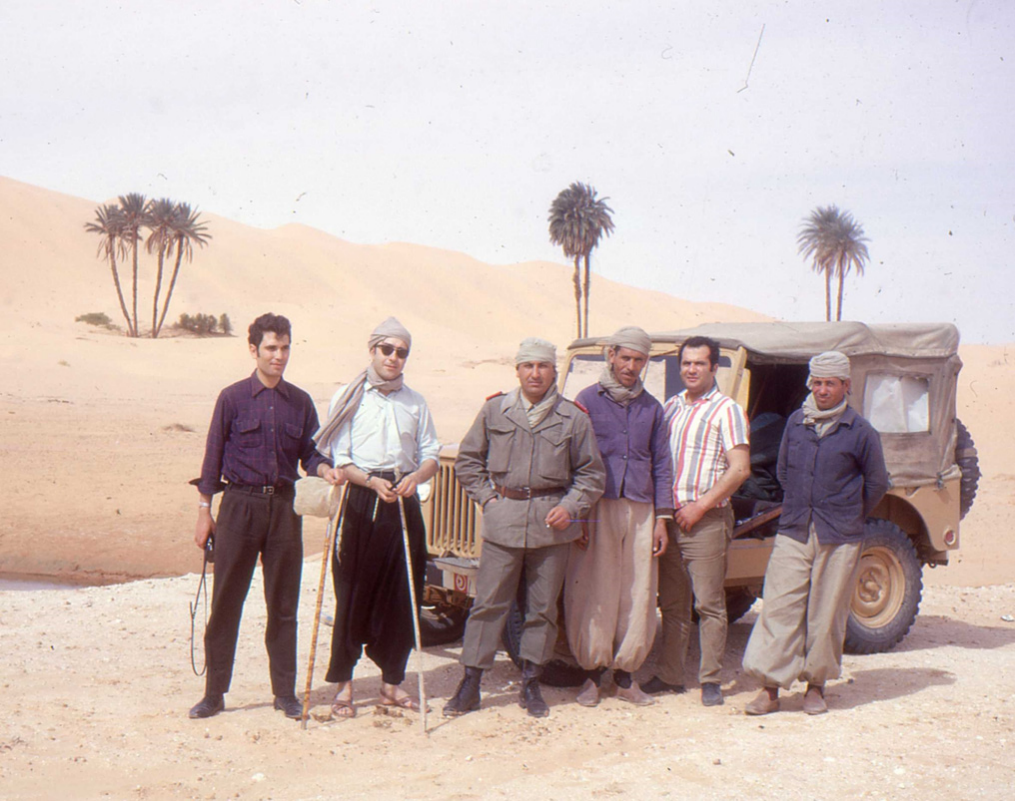
Jean-Antoine Rioux in the Tunisian desert with a team of Tunis Pasteur Institute (1970).

## A major contributor to the study of leishmaniasis

However, from the 1960s, the dominant theme of J.A. Rioux’s work was the area that enabled him to express the fullness of his talents and earned him international recognition: the in depth study of the pathogenic complex of leishmaniasis, a major public health problem in the whole Mediterranean region in particular. Three epidemiological types of leishmaniasis were and are still present in this area: the zoonotic visceral form due to *Leishmania infantum,* the anthroponotic cutaneous form due to *L. tropica* and the zoonotic cutaneous form due to *L. major*.

In France, *L. infantum* was already known in humans and dogs in the South of France from the Spanish to the Italian borders. However, in the peri-Mediterranean foci, the ‘true’ vectors remained to be discovered. The presence of abundant sand fly populations led him to address some of the major issues that are fundamental in parasitology such as *Leishmania* behavior in vectors, vicariant vectors (respective roles of usual *vs.* accidental vectors) and the systematic and eco-physiological specificity of the vector/vertebrate and vector/parasite pairs. Finally, the research conducted in active leishmaniasis illustrated particularly well the “eco-epidemiological” approach dear to the neo-Hippocratic School of Montpellier University. From the beginning of the 1960s, under the auspices of INSERM, a research project on leishmaniasis epidemiology was initiated in the French Mediterranean area. The project included the study of four components: ecology of the parasite cycle actors, ecology of transmission, identification of leishmaniasis risk factors at the spatiotemporal and population levels, and proposals for “integrated control” adapted to each cycle and to each focus. The geographical area of the study ranged from the Cevennes in the North of the focus (Aigoual-Lozère-Espinouse) to the South (coasts of Camargue and Bas Languedoc). The geographical area was divided into epidemiologically homogeneous strata, defined by “zoning indicators” such as altitude, phyto-ecology, and bioclimatic data. In each stratum, simultaneous sampling of the organisms involved in the cycle was carried out: vectors (*Phlebotomus* spp.), reservoirs (domestic and wild Canidae) and parasites (*Leishmania* spp.). During the three decades of this program funded by the INSERM and CNRS, four features highlight this research particularly well. The first was the ecological analysis of the sandfly vectors, involving systematic identification of species, development of trapping techniques to establish their relative densities by biotope, spatial dispersion, trophic behavior and physiological age; the detection of natural infections and intra-vector dynamics of experimental infection; and finally the establishment of laboratory breeding. The second feature was the analysis of disease distribution and prevalence in the canine population by immuno-fluorescence assays. The third was the search for wild animal reservoirs, for example in foxes or rodents. And last but not least, the identification, classification and phylogenetic taxonomy of *Leishmania* based on phenetic, particularly isoenzymatic, analysis. In the Cevennes, one of the main results was the demonstration that the vector role was usually assumed by *P. ariasi*, and not by the locally more abundant species, *P. perniciosus*. The installation and persistence of *L. infantum* was shown to be based on the existence of bioclimatic conditions favorable to *P. ariasi*. One of the great advances, indeed, of this research, was the use of phyto-ecological analysis, where the vegetation layers and bioclimatic variables were essential witnesses of the presence of the vector. These concepts, logically arising from the multidisciplinary knowledge (botany, systematics, and eco-epidemiology) of the eminent naturalist that Jean-Antoine Rioux was, constituted a major breakthrough in the knowledge of the leishmaniasis eco-epidemiology [5,13]. Subsequently, these conclusions could be used to analyze many peri-Mediterranean foci in the Maghreb, Middle East and South America. The natural infection of the vectors was detected from samples taken from areas where canine disease was detected. On this occasion, the delicate techniques of sterile dissection and cultivation of the parasite directly from sandflies were developed in the field. These results were of great interest for the identification of the “true” vectors of *Leishmania* in numerous Mediterranean foci: *L. infantum* (Pyrénées-Orientales, Spain, Algeria, Syria, Crete), *L. donovani* (Syria), *L. tropica* (Morocco) or *L. major* (Morocco). The trophic preferences of *P. ariasi* were determined, demonstrating exophagous and anthropophilic behavior. At the same time, the cynophilic preference of this species was demonstrated, as dogs held on a leash near the bivouacs were regularly targeted. The identification of blood meals completed these results. Considered as unable to fly long distances, the role of sandflies in the dissemination of leishmaniasis was thought to be negligible. Nonetheless, several “mark-release-recapture” experiments unambiguously established that, in Languedoc, dogs were not solely responsible for long-distance transport of *L. infantum* but shared this role with the vector *P. ariasi*. A survey was also performed on the vertebrate reservoir to establish the regional prevalence of canine leishmaniasis in the Languedoc-Roussillon area, in comparison to the distribution of the vector *P. ariasi*, and to track possible vulpine or murine reservoirs. In addition, the contamination of a healthy dog through the blood meal of infested sandflies was carried out at the end of the 1970s, definitively establishing the complete *L. infantum* cycle in a laboratory. To confirm the hypothesis of Percy Cyril Claude Garnham on the possible sylvatic origin of *L. infantum*, an investigation was conducted on the spontaneous and experimental infection of the red fox (*Vulpes vulpes*). Spontaneous infection was detected as early as 1968. The experimental infestation was carried out on two foxes bred in a non-endemic region. Subsequently, foxes infected by *L. infantum* were found in several southern European countries. Therefore, in the area of *L. infantum*, the cycle of the parasite could be regarded as primitively sylvatic (Garnham’s “primary cycle”), with a wild canid as a reservoir. Secondarily, this cycle was anthropized through a passage to the domestic dog (Garnham’s “secondary cycle”). An intermediate stage was readily imagined, involving both wild and domestic hosts (Garnham’s “primary-secondary cycle”). Finally, the human host usually remains a “parasitic dead-end” because leishmaniasis has a low prevalence in human populations and because sandflies cannot readily be infected directly by biting humans (except when co-infected with HIV). Finally, the study completely met its objectives, entirely elucidating the zoonotic cycle of *L. infantum*, which then appeared as an excellent model of parasitic ecology. Many basic but novel concepts had been developed during this research project: pathogenic complex, landscape epidemiology and phyto-ecological zoning, compartmentalized cyclic system, and multifactorial and spatial-temporal evolution of foci (changes in climate and socio-economic conditions). Moreover, for the first time in field parasitology, the concept of a transdisciplinary approach had proven its efficiency [13].

**Figure 4:**
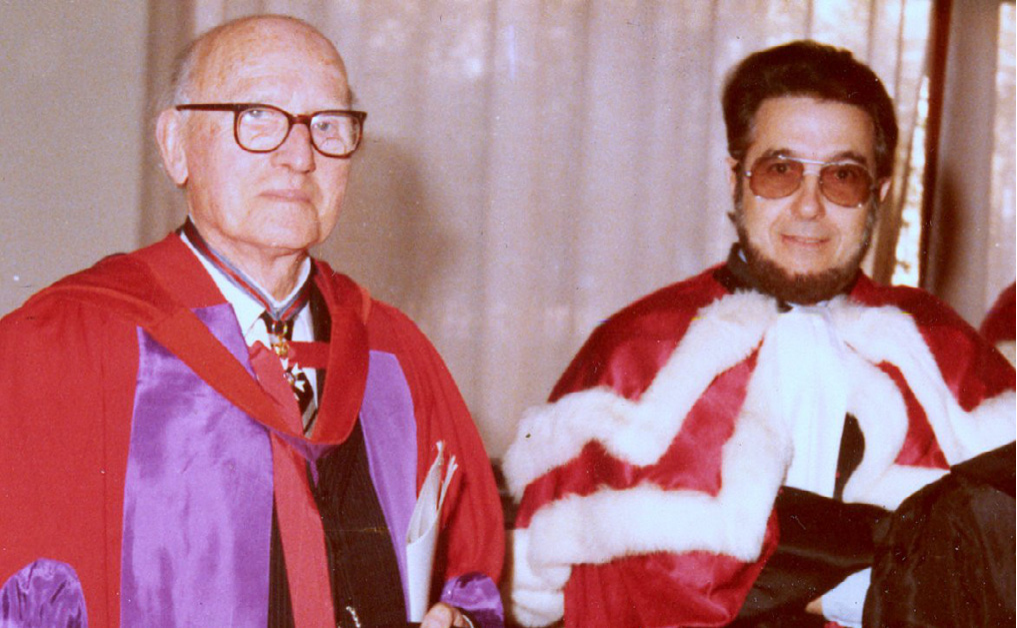
Jean-Antoine Rioux with Percy Cyril Claude Garnham.

Nevertheless, the precise identification of the pathogen, a fundamental element, was lacking. *Leishmania* species are morphologically indistinguishable but show quite different nosological and epidemiological features. Therefore, to further complete the “bio-geographical” analysis of transmission foci, a rigorous and unmistakable identification of circulating parasite populations was required. Morphologically identical, parasites could not be distinguished in culture, as is the case for bacteria, for example. Approaches based on inoculation in animals did not lead much further than those using monoclonal antibodies. However, a new tool based on isoenzyme analysis made it possible to clarify the systematics of the large *Leishmania* genus. Immediately after the publication of this technique, the most robust method for the time, by Michael Chance [1], Jean-Antoine Rioux developed it, with the help of Francine Pratlong, to make Montpellier the world reference center in this field. The acronym MON, for Montpellier zymodeme, remains until today a WHO recognized reference for the classification of *Leishmania* strains and species. Thus, for a decade, using this novel and rigorous tool, his team deepened the taxonomy and systematics of *Leishmania*, culminating in 1990 with the publication of a complete revision of the genus, which still remains the reference today, with more than 500 citations [9]. An “International Center of cryopreservation, enzymatic identification and taxonomic study of *Leishmania*” was also created, which, without going into details, consisted of four interconnected units devoted to cultures, preservation of cryostabilates, identification and classification. Finally, a biobank of isolates was created, supported by the CNRS and officially recognized by the WHO. When Jean-Antoine Rioux retired, nearly 2,500 strains of *Leishmania* were preserved and rigorously identified and designated by official codes (LEM and WHO). These expert activities led to the creation in 1998 of the National Reference Center for Leishmaniasis by Jean-Pierre Dedet, his successor. However, long after his retirement, taxonomy and systematics remained Jean-Antoine Rioux’s preferred disciplines and, until his last days, he continued his studies and deepened his thoughts on the topic and continued to publish on systematics, epistemology, and the history and philosophy of science. Open-minded and strongly interested in the advances of molecular biology, he remained hopeful that a definitive classification of *Leishmania*, bringing together the inputs of isoenzymatic phenotypes and DNA data would be developed.

**Figure 5:**
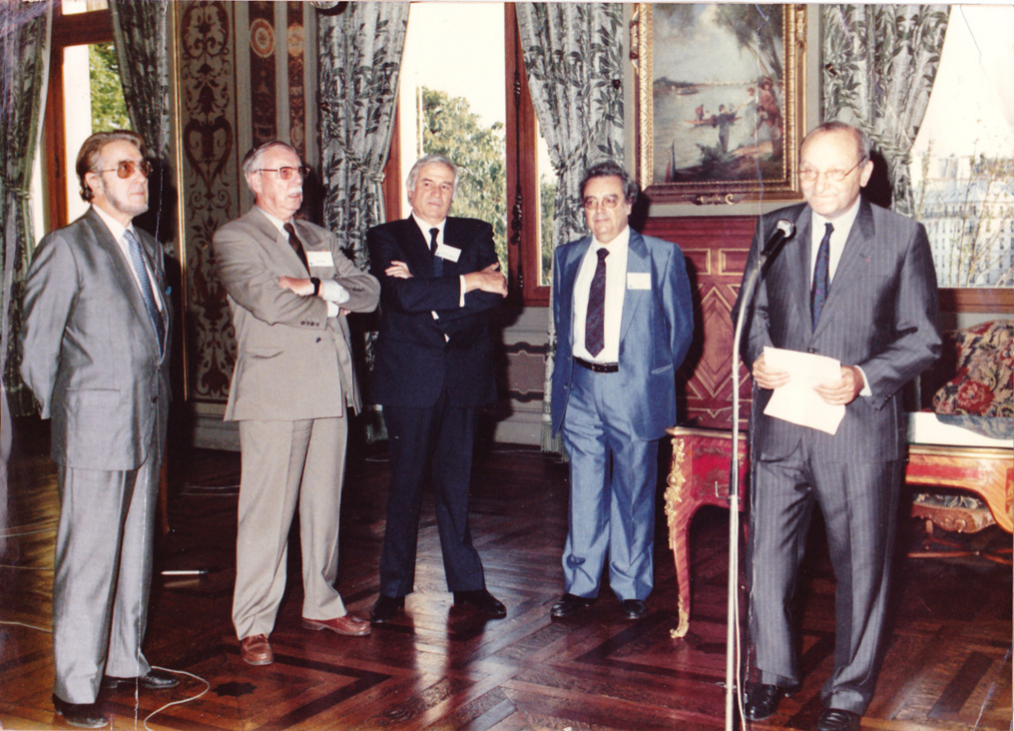
ICOPA Paris 1990. Official reception in Paris Townhall. From left to right: Jean-Antoine Rioux, Jean-Marie Doby, André Aeschlimann (president of the World Federation of Parasitologists), René Houin & Adrien Bedossa (deputy mayor for International Affairs).

The study of two nearby foci, Corsica and Catalonia, confirmed the validity of the concepts implemented in the Cevennes. The first was known to house enzootic canine leishmaniasis and sporadic cases of human visceral leishmaniasis. The canine infestation was systematically searched for in about 30,000 animals, and the *Leishmania* species cultivated and identified. At the same time, sandflies were screened throughout the island. The results were very similar to those found on the French mainland. These studies in Corsica enabled fruitful and frequent exchanges with the Italian team of Ettore Biocca and Alberto Coluzzi working in neighboring Sardinia. However, in Catalonia, the *Leishmania* “pathogenic complex” was different from that of Languedoc by the relative abundance of *P. perniciosus* compared to *P. ariasi*, and by the presence of *P. sergenti*, a recognized vector of *L. tropica*. A few years later, strictly cutaneous human leishmaniasis, known as “autochthonous oriental sore”, proved to be common in the Pyrénées-Orientales department of France. Moreover, the enzymatic taxonomy, developed in the meantime, linked these lesions not to *L. tropica*, as many assumed, but to *L. infantum*. More importantly, besides the presence of the classic zymodeme MON-1, several other zymodemes were detected, some unknown until then such as MON-29, MON-11 and MON-33, particularly related to cutaneous forms. Several field surveys, on both sides of the French-Spanish border, clarified the characteristics of this focus. As with the Cevennes survey, the prevalence of canine leishmaniasis was established. The canine infestation was confirmed by the isolation of 27 strains of *Leishmania*, all reported to belong to the MON-1 zymodeme. In addition, 13,635 sandflies were harvested by adhesive traps following different transects and identified as belonging to the genera *Phlebotomus* and *Sergentomyia*. The prevalence in humans was determined using the Montenegro test which was carried out in 718 children. Statistical analysis showed a good correlation among the altitudinal prevalences of the three components of the leishmanial complex. These prevalences were highest between 300 and 600 m above sea level and lowest below 150 m and above 600 m. In the field, the maximum prevalence corresponded to the *Quercus ilex* and *Q. pubescens* mixed forest level which was then considered in the Cevennes as a “risk zone”. Dissections of *P. ariasi* and *P. perniciosus* carried out in the vicinity of Céret (Pyrénées-Orientales) led to the isolation and identification of two of the zymodemes (*L. infantum* MON-1 and MON-29) already observed in autochthonous human cases of cutaneous leishmaniasis. The same results were obtained in a comparable survey in Spanish Catalonia. However, the search for *Leishmania* in small mammals (such as *Rattus rattus,* a species implicated in the *L. infantum* cycle in Italy) remained negative.

The successful application of the concepts imagined by Jean-Antoine Rioux on the circulation of *Leishmania* parasites to a geographically close but structurally different focus, confirmed their validity. Their universality was then proven by their use for a large number of other foci and by their popularity in the international *Leishmania* community. Tirelessly challenged and refined by Jean-Antoine Rioux, they remained the basis of the work that he never stopped publishing until his last months. In the same way, the Center for cryopreservation, enzymatic identification and taxonomic study of *Leishmania*, the founding element of the National Reference Center for Leishmaniasis, was the subject of fruitful studies throughout the whole career of its designer, and was passed on to his successor at his retirement. Led by a team of skillful scientists and technicians, constantly improving, it was an essential tool of the work carried out by what had become “the School of Montpellier” and by all those who collaborated with it, from France or all over the world. Jean-Antoine Rioux detailed the results obtained in the study of the ecology of leishmaniasis in the South of France in 22 papers published in the *Annales de Parasitologie Humaine et Comparée* and *Parasite* from 1967 to 2013 [4,15]. Many research programs were conducted in Europe (France, Spain, Italy, Cyprus), North Africa (Morocco, Algeria, Tunisia, Egypt), the Middle East (Yemen, Syria, Iraq, Oman), Saharan Africa (Chad, Senegal) and Latin America (Colombia, Ecuador) and their analysis confirmed the value of all the concepts developed by J.A. Rioux. It is not possible to detail here all these collaborative research initiatives. Let us illustrate one of them, in Morocco, which lasted for 30 years. It was made possible by an exemplary cooperation which resulted in the understanding of a very complex situation and the implementation of control and prevention measures.

In Morocco, as in most peri-Mediterranean countries, leishmaniasis has always been an important public health problem [11]. Visceral or cutaneous, zoonotic or anthroponotic leishmaniasis are present in Morocco from the North to the South, from the Rif cedar woods to the Anti-Atlas palm groves, with several species involved: *L. infantum*, *L. major* and *L. tropica*. This research program was carried out by the Moroccan Ministry of Health (Directorate of Epidemiology) and the Department of Parasitic Ecology (University of Montpellier1/CNRS) with participating parasitologists from the Reims and Barcelona Faculties of Pharmacy, from Medellin, Colombia (Faculty of Sciences) and Trujillo, Venezuela (Faculty of Medicine). Several non-academic organizations supported the research: French, Moroccan and Spanish Ministries of Cooperation, Ministry of Foreign Affairs, WHO, EU, CNRS, and INSERM. The survey began in 1970 by the taxonomic and chorologic study of sandflies. Nineteen species were identified, including several so far unknown species in Morocco and at the world level. The densities of each species, expressed in frequency classes per station, were processed by factorial analysis of correspondences. Using thematic maps, each station could be related to its phyto-ecological level and bioclimatic zone. Each of the species considered a vector of *Leishmania* was accurately located. Once again, the pre-eminence of the climatic factor in the geographical distribution and abundance of the vectors and, consequently, in the distribution and strength of infection of the foci appeared indisputably. But beyond the entomological survey, a long path remained before it was possible to achieve the structural and dynamic knowledge of the various Moroccan foci. Two decades were required to evaluate the epidemiology of visceral and cutaneous leishmaniasis in Morocco. Most of the local epidemiological cycles were determined after enzymatic identification of *Leishmania* species in vectors, animal reservoirs and humans. For each focus, potential risks were assessed, including those related to potential climate changes. Finally, “integrated control” was implemented in several pilot sites. Led by the Moroccan Ministry of Public Health, these operations were enhanced through the establishment of an epidemiological monitoring observatory. Each of the three *Leishmania* species found in Morocco was specifically studied. The first Moroccan outbreak of human cutaneous leishmaniasis due to *L. major* was reported at the end of the 1970s, in the south of Anti-Atlas, in the district of Tata. The 51 isolates obtained from human samples were identified as *L. major* MON-25, a zymodeme observed throughout Maghreb, from Morocco to Libya. A Gerbillidae reservoir and a vector of the subgenus *Phlebotomus* were also identified. Out of a total of 484 rodents, only *Meriones shawi grandis* was infected with *L. major* MON-25 (12 isolates identified with an overall prevalence of 14%). The infection, strictly cutaneous, could persist for several years without apparently affecting the animals. However, an intense parasitic diffusion was observed at the end of the course. This long skin tolerance, followed by a short phase of high release, gave *Meriones shawi* the quality of the “real reservoir” of *L. major* [7]. Its optimum biotope was made up of circular pits dug by man in the vicinity of the dwellings. Originally used for the manufacture of raw clay bricks, these excavations were subsequently used as dumps and latrines. Becoming detritivore and coprophagous, the rodents behaved like a dangerous commensal. At the same time, vector infection was identified 70 km east of Tata. A total of 1,673 *Phlebotomus* sp. females were dissected and only *P. papatasi* was found carrying *L. major* MON-25. To control the disease, it was therefore proposed to physically destroy *M. shawi* biotopes, to use rodenticide campaigns, and to regularly monitor the density of rodent populations. In parallel, human mass screening and treatments were recommended to accelerate healing of the lesions. In southern Morocco, the range of the other species, *L. tropica*, covered 40,000 km^2^. On the northern slope of the Upper Atlas, the “risk zone” extended from east to west, in a continuous fringe, between an altitude of 500 and 1,000 m above sea level. This area corresponds to the semi-arid layer biotope of the Barbary thuja. In most inventoried sites, human cutaneous leishmaniasis was reported on the “rural mode”, i.e. hypoendemic, dispersed and unstable (mean incidence 5%). The importance of enzymatic polymorphism of *L. tropica* in the Upper Atlas focus (MON-102, MON-107, MON-112, MON-113, MON-122, MON-123) and the distribution of zymodemes in three distinct phenetic sub-groups, suggested successive inputs of Oriental origin, rather than *in situ* differentiation from a Moroccan ancestor. Diffuse and pseudo-lepromatous infiltrative forms, already observed in Yemen and Syria among the elderly, were also observed in Morocco in the earliest surveys. These lesions, with a prolonged course and rich in amastigotes, constituted an important source of parasites available to the vector. In these specific cases, *L. tropica* cycle appeared to be of an anthroponotic nature. In the Upper Atlas foci, as in the north of Syria, the domestic dog was seen with strictly cutaneous lesions due to *L. tropica*. However, the animal did not appear to constitute a “real” reservoir, despite its high infestation rate (12%) and its contamination by MON-102 and MON-11 zymodemes also observed in humans. In dogs, the skin lesions were in fact discreet and transient and no cases of generalization to viscera were observed. It should be noted that in *L. tropica* Moroccan foci, the dog was often parasitized by *L. infantum* MON-1. Furthermore, *P. sergenti*, a recognized vector of *L. tropica*, was abundant in all Moroccan foci. The isolates obtained from the vectors were typed as MON-102, MON-107, MON-122 and MON-123; the two latter zymodemes had never been observed in humans or dogs. As for *L. major* and *P. papatasi*, the period for transmission of *L. tropica* by *P. sergenti* was done at the end of the hot season. The recommendations to control cutaneous leishmaniasis due to *L. tropica* did not include insecticide control because of the hypo-endemic nature of the disease, its “dispersed” distribution, difficulties of access to the contaminated douars and the cost of operations. Therefore, control was based on active case detection and subsequent treatment of lesions. This strategy, which was successfully tested in the Tanat district, could be generalized to all foci. In Morocco, as in the other Maghreb countries, human and canine visceral leishmaniasis had been known for several decades. If the distribution and importance of zoonotic foci were well known, the identification of the “true” vectors and the existence of human skin forms remained debated. Most of them came from moderate-altitude areas of the Rif and of the Atlas. The involved species was *L. infantum* MON-1. However, *L. infantum* was not only responsible for visceral forms. In 1990, about 100 cases of human cutaneous leishmaniasis were detected in the district of Taghjicht and were due to *L. infantum* MON-24, a zymodeme usually responsible for sporadic cases. The entomological investigation showed that *P. longicuspis* s.l. was the only sandfly of the subgenus *Larroussius* present at the site infected by promastigotes (strain not isolated). Finally, the presence of *L. infantum* was proven on almost all the Moroccan territory. This extensive distribution was explained by the presence of three vectors, *P. ariasi*, *P. perniciosus*, *P. longicuspis*, whose bioclimatic preferences and consequently geographical distributions differed. *P. perniciosus*, was predominantly present in the semi-arid/sub-humid Rif and the Middle Atlas. *P. ariasi*, accompanied it in the wet/sub-humid zones of Rif and on the northern slopes of the Upper Atlas. *P. longicuspis* and its possible twin species, preferring arid/peri-arid zones, cohabited with the previous species, but were observed alone in the arid, warm and Saharan zones. In other words, in Morocco, the North-South succession of vectors and their association with different bioclimatic zones allowed *L. infantum* to cover the whole territory.

## Conclusions

In the course of these investigations, many new concepts and techniques were developed and clarified such as analysis of temporo-spatial risk, phytogeographical cartography, role of anthropization (impoverishment, desertification) in the emergence of new foci, “integrated control” and, recently, the role of climate change. We must also stress the quality of the scientific and personal relationships established with the countless teams of the countries in which these programs were carried out: the striking enthusiasm of Jean-Antoine Rioux, his charisma and his encyclopedic knowledge compelled admiration of all researchers. His field missions, particularly in the Cevennes, also attracted a number of foreign scientists, on whom he left an indelible imprint. Two international symposia, held in Montpellier in 1974 (under the auspices of INSERM) and in 1984 (under the auspices of CNRS and WHO), crowned these two decades of research in eco-epidemiology and taxonomy of *Leishmania*. They brought together more than 100 participants from all over the world (UK, USA, Belgium, Germany, Italy, Spain, Portugal, Iran, Israel, USSR, Brazil, Venezuela, Colombia, Morocco, Algeria, Tunisia, and others), among whom were prominent experts either in epidemiology or in biochemical and molecular typing. The proceedings of these two symposia are still today the most important references in the field [5,8]. J.A. Rioux was a member of numerous scientific societies and academies, among which the *“Académie Royale de Médecine de Belgique*”, “*Académie Royale des Sciences d’Outre-Mer* (Brussels), and the *“Académie des Sciences et Lettres de Montpellier”*. Among the many awards received in recognition of his work, let us quote the Marchaux prize of the French National Academy of Medicine (1969), the Silver medal of the French Society of Parasitologists (1999), and a special award presented during WorldLeish 5 (Brazil, 2013) *“in recognition of his support and estimable contribution to the field of leishmaniasis*”, as well as a posthumous tribute in WorldLeish 7 (Spain, 2017). The results from Jean-Antoine Rioux’s research presented here are obviously far from being the only ones this tireless researcher obtained. He published or participated in the publication of more than 500 papers, to which must be added books and the supervision of many PhDs. To use the words of one of us (Patrick Bastien, his current successor at the head of the Parasitology Department in Montpellier University): *“Mr. Rioux was one of those men whom one crosses only too seldom and who cannot be forgotten once you knew him*”. President of the French Society of Parasitology from 1982 to 1999, Jean-Antoine Rioux brought the society to the rank it currently occupies, in particular by providing it with modern bylaws. He was able to motivate the members to organize the 7^th^ International Congress of Parasitology (ICOPA7) in Paris in 1990, which brought together more than 2,500 participants. To conclude, we should add that this brilliant teacher, an impeccable French language writer, was also a man of culture, and that he never ceased to be, for those who accompanied him throughout these years, a friend equally unforgettable.

## Conflicts of interest

The authors declare no conflict of interest

## A note about the collections of Jean-Antoine Rioux

All collections made by Jean-Antoine Rioux have been transferred to the laboratory of Parasitology of Faculté de Pharmacie, Université de Reims Champagne Ardenne, Reims, France, where they will be curated by Prof. Jérôme Depaquit.

## List of taxa described by Jean-Antoine Rioux

*Aedes coluzzii* Rioux, Guilvard, & Pasteur, 1998 [Diptera, Culicidae]

*Anopheles rufipes seneveti* Rioux, 1960 [Diptera, Culicidae]

*Carouxella* Manier, Rioux & Whisler, 1961 [Trichomyceta]

*Carouxella scalaris* Manier, Rioux & Whisler, 1961 [Trichomyceta]

*Coelomomyces grassei* Rioux & Pech, 1960 [Blastocladiales, Coelomomycetaceae]

*Coelomomyces raffaelei* Coluzzi, Rioux, 1962 [Blastocladiales, Coelomomycetaceae]

*Coelomomyces tuzetae* Manier, Rioux, Coste & Maurand, 1970 [Blastocladiales, Coelomomycetaceae]

*Culicoides agathensis* Callot, Krémer & Rioux, 1963 [Diptera, Ceratopogonidae]

*Culicoides caucoliberensis* Callot, Krémer, Descous & Rioux, 1967 [Diptera, Ceratopogonidae]

*Culicoides haranti* Rioux & Descous, 1959 [Diptera, Ceratopogonidae]

*Culicoides maritimus paucisensillatus* Callot, Krémer & Rioux, 1963 [Diptera, Ceratopogonidae]

*Leishmania (Leishmania) killicki* Rioux, Lanotte & Pratlong, 1986 [Kinetoplastida, Trypanosomatidae]

*Phlebotomus (Larroussius) chadlii* Rioux, Juminer & Gibily, 1966 [Diptera, Psychodidae]

*Phlebotomus (Larroussius) mariae* Rioux, Croset, Léger & Bailly-Choumara, 1974 [Diptera, Psychodidae]

*Phlebotomus (Paraphlebotomus) chabaudi* Croset, Abonnenc & Rioux, 1970 [Diptera, Psychodidae]

*Rubetella inopinata* Manier, Rioux & Wisler, 1961 [Trichomyceta]

*Stempellia tuzetae* Rioux, Tour & Croset, 1971 [Microsporidia, Nosematidae]

*Trychophyton vanbreuseghemii* Rioux, Jarry & Juminer, 1964 [Fungi imperfecti]

## List of taxa named in honor of Jean-Antoine Rioux

*Adelina riouxi* Levine, 1977 [Coccidia, Adeleidae]

*Amphinomia riouxii* Quézel, 1959 [Rosales, Leguminosae]

*Culicoides riouxi* Callot & Krémer, 1961 [Diptera, Ceratopogonidae]

*Empidomermis riouxi* Doucet, Laumont & Bain, 1979 [Nematoda, Mermithidae]

*Hystrichopsylla talpae riouxi* Beaucournu & Rosin, 1977 [Siphonaptera, Hystrichopsyllidae]

*Longicollum riouxi* Golvan, 1969 [Acanthocephala, Pomphorhynchidae]

*Phlebotomus (Paraphlebotomus) riouxi* Depaquit, Léger & Killick-Kendrick, 1998 [Diptera, Psychodidae]

*Pseudorhabdosynochus riouxi* (Oliver, 1986) Kritsky & Beverley-Burton, 1986 [Monogenea, Diplectanidae]

*Riouxgolvania* Bain & Brunhes, 1968 [Nematoda, Muspiceidae]

## À la mémoire du Professeur Jean-Antoine Rioux (1925-2017)

Né à Naucelle (Aveyron), d’une famille limousine solidement ancrée dans le terroir, Jean Antoine Rioux a passé son enfance au Vigan, dans le milieu très typé des Cévennes : c’est sans aucun doute là qu’il a acquis la passion de l’étude de la nature, qui a sous-tendu toute son activité. Cette fascination l’a amené à approfondir l’étude de la Botanique, compétence qui lui a valu, beaucoup plus tard (1977-1993), d’être nommé Directeur du Jardin des Plantes de Montpellier [12]. Il s’y est consacré pendant de nombreuses années, avec talent et sans relâche, préservant et enrichissant les collections vivantes, rénovant les serres, promouvant son image et assurant sa protection par un classement au double titre des « Sites et paysages » (1982) et des « Monuments historiques » (1992). À cette culture initiale s’est ajoutée, pendant ses études à Montpellier, une vocation médicale tout aussi puissante. Brillant étudiant, il est major à l’Internat en 1951, puis chef de Clinique des hôpitaux de Montpellier, titulaire du CES de dermatologie, mais aussi de celui de pneumo-phtisiologie. Il trouve très tôt le domaine qui lui permettra de se réaliser, en entrant dès 1952 au Laboratoire d’Histoire Naturelle Médicale dirigé par le Pr. Hervé Harant, brillant naturaliste et parasitologue à la vaste culture scientifique. Il acquiert le CES de Parasitologie, le diplôme de Médecine exotique, et il poursuivra toute sa carrière dans cette structure devenue Laboratoire de Parasitologie, puis Laboratoire d’Écologie médicale et Pathologie parasitaire. Il en fera un outil remarquable, reconnu et soutenu par les instances de la recherche, permettant de conduire des travaux matérialisés par plus de 500 publications.

### Ses premiers travaux entomologiques

Dans les Facultés de Médecine françaises, l’épidémiologie du paludisme a toujours été l’un des grands chapitres du *cursus* de Parasitologie. Le jeune enseignant s’est attelé à transformer le sujet de cet enseignement en activité de recherche. Au cours de la préparation de sa thèse sur les *«* *Culicidae du Midi méditerranéen* *»,* il avait acquis une solide formation entomologique. Il l’a appliquée à l’étude du paludisme en Languedoc-Roussillon, proposant le concept de « paludisme instable ». Ce dernier sera repris plus tard, à l’occasion de plusieurs missions écoépidémiologiques au Nord-Tchad (Borkou-Ennedi-Tibesti). La persistance, même temporaire du parasite chez des oasiens autochtones et surtout chez des enfants sédentaires, témoignait d’une transmission *in situ*, c’est-à-dire de la présence effective de vecteurs. De fait, il trouva des larves du complexe *Anopheles gambiae* dans les gîtes de débordement des mares de Faya-Largeau et un an plus tard les adultes étaient capturés dans une habitation de l’oasis. À l’occasion de ces prospections, 25 espèces culicidiennes ont été identifiées, apportant d’importantes précisions biogéographiques. Dans le Midi méditerranéen, il consacra aussi une étude à l’autogenèse présente, de manière variable, chez l’espèce halophile sub-littorale *Aedes (Ochlerotatus) detritus*. Cette variabilité évoquait soit un simple polymorphisme génétique, soit une véritable ségrégation populationnelle (espèce cryptique). L’établissement des fréquences alléliques isoenzymatiques, chez des adultes issus d’un même gîte larvaire, confirmait la deuxième hypothèse (collaboration avec Nicole Pasteur). Cette espèce jumelle a été décrite sous le binôme *Aedes (Ochlerotatus) coluzzii* Rioux, Guilvard, Pasteur [10]. *A. coluzzii* s’entretenait sans difficulté en insectarium, alors que pour obtenir une descendance fertile avec *A. detritus* s. st., il est nécessaire d’utiliser la fécondation artificielle. Cette compétence en Entomologie l’a aussi amené à une importante enquête écologique sur *Leptoconops irritans*, un Ceratopogonidae particulièrement agressif en zones littorales, de la Camargue au Roussillon [3]. Cette action avait pour objectif d’identifier les biotopes larvaires et d’en préciser la dynamique saisonnière. Un transect d’échantillonnage a été réalisé durant deux années en Moyenne Camargue. Les résultats ont permis de préciser la situation et le fonctionnement des sites d’émergence de *L. irritans*, les phytocénoses les plus productives étant constituées par les groupements à Chénopodiacées vivaces qui forment l’essentiel des écosystèmes halophiles de Moyenne et Basse Camargue. C’est aussi sur ces bases qu’a été proposée la « lutte raisonnée » contre les « moustiques- nuisances » ou « démoustication » (au sens de l’inventeur du mot, H. Harant). Le concept de « nuisance » concerne des espèces hématophages responsables d’inconfort et non de transmission d’agents pathogènes (vecteurs). Pour agir, il est nécessaire de disposer de données autoécologiques concernant tous les stades de développement de l’insecte. Cette situation est bien différente de celle des maladies à transmission vectorielle, où le vecteur n’est que la composante entomologique du complexe pathogène, qui comprend également les hôtes vertébrés (dits réservoirs), dont l’homme, et le pathogène lui-même. Il faut alors intervenir sur des systèmes cycliques compartimentés, dont le contrôle réclame des stratégies opérationnelles bien différentes (lutte intégrée). Cette situation a été développée dans les travaux sur les leishmanioses qui sont présentés plus loin. Telle qu’elle a été définie initialement, la démoustication en Languedoc-Roussillon intéressait quatre espèces fortement nuisantes : *Culex pipiens*, *A. caspius*, *A. detritus* et *A. coluzzii*. L’opération, initiée par l’Entente Interdépartementale pour la Démoustication (EID Languedoc-Roussillon, créée en 1958), a été menée par la Mission interministérielle d’aménagement touristique du littoral du Languedoc-Roussillon (« Mission Racine ») créée en 1963. Dans les zones humides sublittorales, à forte nuisance culicidienne, la priorité était donnée à la lutte anti-larvaire, la lutte anti-adulte n’étant utilisée qu’en cas d’échec de celle-ci ou pour assurer la protection des stations touristiques à l’égard des nuées d’adultes issus des sites voisins non démoustiqués (ex. réserves naturelles). C’était le cas de la Grande Motte et de Port-Camargue, cités littorales régulièrement envahies à partir de la Grande et de la Petite Camargue. En quelques années, l’application en vraie grandeur de la stratégie ainsi définie a permis de réduire la nuisance rurale de manière significative, tout en minimisant les effets négatifs sur les faunes et flores non-cibles. Simultanément, la lutte contre l’espèce domestique *C. pipiens* a été basée sur le dépistage et la cartographie des biotopes larvaires urbains et périurbains (caves, égouts, fosses septiques, bassins d’ornement, réceptacles d’eau de pluie, récipients divers). Dans la plupart des cas, le comblement des gîtes (par exemple les vides sanitaires), leur couverture par des billes de polystyrène (par exemple dans les caves inondées), voire leur suppression définitive (par exemple les récipients abandonnés) ont suffi à contrôler la nuisance. Les insecticides n’étaient utilisés qu’en dernier recours, en raison de phénomènes de résistance. Dans ce type de lutte, l’éducation et la sensibilisation du public devaient jouer un rôle majeur. Conçue il y a plus d’un demi-siècle, la démoustication du littoral Languedoc-Roussillon est restée d’une remarquable efficacité et l’exemple d’une intégration réussie entre le politique, le scientifique et l’opérationnel [14]. Elle le doit à la compétence des équipes qui ont été réunies par le concepteur et ses successeurs. Cette structure est devenue un centre pédagogique de premier plan, tant pour les étudiants français et étrangers que pour les chercheurs confirmés. D’ailleurs, dès les succès des années 60, la méthode a été importée dans d’autres régions françaises, côtières ou continentales (par exemple EID Atlantique, Rhône-Alpes, Alsace). En Guadeloupe, toujours en collaboration avec l’EID, une carte phyto-écologique des gîtes de mangroves était levée à l’échelle du 1/25000ème. Enfin, plusieurs pays étrangers ont fait appel à la structure montpelliéraine : Tunisie, Maroc, Espagne, Grèce, Chypre, Canada.

### Peste et Bilharziose

Avant d’aborder les travaux sur les leishmanioses, et en introduction aux études menées sur les complexes pathogènes, il faut citer deux programmes ciblés non sur des vecteurs mais sur des réservoirs de parasites. Ils sont relativement limités dans le temps, mais ils ont apporté des résultats hautement significatifs. Le premier, une œuvre de jeunesse, a été mené avec Yves Jean Golvan et l’Institut Pasteur de Téhéran en 1960. Il portait sur les réservoirs de la peste enzootique au Kurdistan iranien, des Rongeurs Gerbillidae (*Meriones* spp.). Cette étude, conduite sur un cycle biologique annuel, a permis d’identifier les biotopes des principales espèces impliquées (localisation spatiale des peuplements, caractéristiques géomorphologiques des terriers, préférences trophiques spécifiques, cartographie phyto-écologique des zones à risque) et de définir les rôles des espèces sensible (*Meriones persicus*) et résistante (*M. vinogradovi*) entre lesquelles se perpétue la circulation du bacille [2]. Au terme de l’enquête, les pratiques agro-pastorales traditionnelles (pâturages extensifs, céréalicultures « en sec ») ont été considérées comme des déterminants majeurs du foyer pesteux. Le second a été conduit sous l’autorité de la Délégation Générale à la Recherche Scientifique et Technique (DGRST), à partir de 1972. Il concernait un projet de lutte raisonnée contre la Bilharziose intestinale en Guadeloupe. Le Pr. Rioux en avait la responsabilité opérationnelle au niveau central. L’opération a nécessité la collaboration de nombreuses équipes de spécialistes : écologues, épidémiologistes, parasitologues, malacologues, hydrobiologistes, sociologues et médecins. Le « bassin versant », avec son réseau hydrographique, ses Mollusques vecteurs et son occupation humaine a constitué l’élément fédérateur (épidémiologie du paysage). Après dix ans de recherches *in natura*, accompagnées d’interventions de caractère intégré (aménagement des cours d’eau contaminés, dépistage et traitement des cas cliniques et des porteurs sains, lutte contre le péril fécal, éducation sanitaire) la transmission anthroponotique a pu être interrompue [6]. Cependant, certains micro-foyers sylvatiques (par exemple dans les mangroves), comportant le Rat noir comme réservoir, sont demeurés actifs (transmission zoonotique). Le maintien de leur surveillance a été recommandé.

### Un contributeur majeur à l’étude des leishmanioses

Sous leur diversité apparente, tous ces thèmes de recherche ont été sous-tendus par les concepts et les méthodes de l’Écologie générale appliqués à l’Épidémiologie traditionnelle. Les travaux réalisés sur ce thème ont conduit à l’individualisation d’une discipline nouvelle, l’éco-épidémiologie, reconnue aujourd’hui par plusieurs instances nationales (CNRS) et internationales (OMS, FAO). Plusieurs ont concerné les seuls organismes d’un cycle parasitaire, qu’il s’agisse des agents infectieux eux-mêmes, de leurs vecteurs ou de leurs réservoirs. D’autres ont traité de recherches plus finalisées, telle que la « lutte raisonnée » contre les Moustiques- nuisances. Un autre enfin a concerné, à partir des années 60, l’ensemble d’un complexe pathogène, les leishmanioses : c’est de très loin le thème dominant de l’œuvre de Jean-Antoine Rioux, celui qui lui a permis d’exprimer la plénitude de ses talents et lui a valu la notoriété internationale. Le sujet s’y prêtait. Les affections correspondantes sévissaient sur l’ensemble de la Région méditerranéenne où trois modalités épidémio-cliniques étaient représentées : la forme viscérale zoonotique à *Leishmania infantum* et les deux formes cutanées : anthroponotique à *L. tropica* et zoonotique à *L. major*.

En France*, L. infantum* était déjà connue, chez l’Homme et le Chien, de la frontière espagnole à la frontière italienne. Dans les foyers périméditerranéens, les « vrais » vecteurs restaient à découvrir. La présence d’une abondante faune phlébotomienne permettait d’aborder certains grands problèmes de parasitologie fondamentale, tels que le comportement des *Leishmania* chez le vecteur ou les problèmes de vicariance vectorielle (vecteur habituel *vs* vecteur accidentel) et de spécificité (systématique et écophysiologique) des couples vecteur/vertébré et vecteur/parasite. Enfin les recherches conduites dans les sites leishmaniens actifs permettaient d’illustrer concrètement la démarche « éco-épidémiologique » chère à l’École néo-hippocratique de Montpellier. Dès le début des années 1960, sous l’égide de l’INSERM, a été engagée une action de recherche sur l’épidémiologie des leishmanioses dans le « Midi » méditerranéen. Le projet comportait quatre volets : l’écologie des organismes du cycle ; l’écologie de la transmission ; l’établissement des « risques leishmaniens », spatio-temporels et populationnels ; la proposition de projets de « lutte raisonnée » adaptés à chaque cycle et à chaque foyer. La zone géographique de l’étude allait des Cévennes au Nord (Aigoual-Lozère- Espinouse) au littoral au sud (Camargue − Bas Languedoc). L’espace géographique était découpé en strates épidémiologiquement homogènes, définies par des « indicateurs de zonage » : altitudinal, phytoécologique, bioclimatiques… Dans chacune, il était procédé à l’échantillonnage concomitant des organismes du cycle : vecteurs (*Phlebotomus* spp), réservoirs (Canidae domestiques et sauvages) et parasites (*Leishmania* spp). Durant les trois décennies de l’action INSERM puis CNRS, quatre temps forts devaient scander la recherche :

- 1°L’analyse écologique des Phlébotomes vecteurs, comportant l’identification systématique des espèces ; la mise au point des techniques de piégeage, en vue de l’échantillonnage des populations imaginales et de l’établissement de leurs densités relatives, par strates et par« milieux » ; le dépistage de l’infestation naturelle et la dynamique intra-vectorielle de l’infestation expérimentale ; la dispersion spatiale ; le comportement trophique et l’âge physiologique ; et enfin les élevages.
- 2°La chorologie et la fréquence de la maladie canine (abondances stationnelles et zonales des taux d’anticorps immuno-fluorescents).
- 3°La recherche de réservoirs sauvages (Renard, Rongeurs).
- 4°L’identification et la classification des *Leishmania* (taxonomie isoenzymatique, phénétique et phylogénétique).

Parmi les résultats majeurs, on peut citer la démonstration que, dans la zone étudiée, le rôle de vecteur habituel était tenu par *P. ariasi*, et non par *P. perniciosus, a priori* pourtant plus abondant. En Cévennes, l’installation et la persistance de *L. infantum* reposaient sur l’existence de conditions bioclimatiques favorables à ce vecteur. Une des grandes avancées, en effet, de ces recherches, fut l’introduction des concepts phytoécologiques, où l’étage de végétation (dit ‘bioclimatique’) constitue un témoin primordial de la présence de l’insecte vecteur. Ces concepts, logiquement forgés à partir des connaissances multidisciplinaires (botanique, systématique, éco-épidémiologie…) du naturaliste éminent qu’était le Pr. Rioux, a profondément marqué l’épidémiologie des leishmanioses [5,13]. Par la suite, ces conclusions devaient être appliquées à l’analyse de nombreux foyers circumméditerranéens, tels ceux du Maghreb et du Proche-Orient, et par d’autres auteurs en Amérique du sud. L’infestation naturelle des vecteurs a été détectée à partir d’échantillons prélevés dans des sites où avait été dépistée la maladie canine. À cette occasion, les techniques délicates de dissection stérile et de mise en culture du parasite, étaient mises au point sur le terrain. Ces recherches devaient revêtir un grand intérêt dans le dépistage des « vrais vecteurs » de *Leishmania* en Région méditerranéenne, qu’il s’agisse de *L. infantum* (Pyrénées-Orientales, Espagne, Algérie, Syrie, Crête), de *L. donovani* (Syrie), de *L. tropica* (Maroc) ou de *L. major* (Maroc). Les préférences trophiques de *P. ariasi* ont été établies, démontrant un comportement « exophage » et « anthropophile ». Parallèlement, la « cynophilie » de l’espèce était constatée : des chiens tenus en laisse près des bivouacs étaient régulièrement attaqués. L’identification des repas sanguins a complété ces résultats. Considérés comme de mauvais voiliers, le rôle de ces vecteurs dans la dissémination était considéré comme négligeable. Plusieurs opérations de « marquage-lâcher-recapture » ont établi sans ambiguïté qu’en Languedoc, le Chien n’était pas le seul responsable du transport de *L. infantum* à longue distance : il partageait ce rôle avec le vecteur *P. ariasi*. Une enquête sur les Vertébrés-réservoirs a également été mise en place pour établir les fréquences zonales de la leishmaniose canine en Languedoc-Roussillon (parallèles à celles du vecteur *P. ariasi*), tenter la transmission Chien-Phlébotome-Chien, et dépister d’éventuels réservoirs sauvages, vulpins ou murins. De la même manière, la contamination d’un chien sain par la piqûre de Phlébotomes parasités a été réalisée à la fin des années 70, établissant de façon définitive le cycle de *L. infantum* en laboratoire. Pour confirmer l’hypothèse de Percy Cyril Claude Garnham sur l’éventualité d’une origine sylvatique de *L. infantum*, une enquête a été menée sur l’infestation leishmanienne, spontanée et expérimentale, du Renard roux, *Vulpes vulpes*. L’infestation spontanée a été dépistée dès 1968. L’infestation expérimentale a été réalisée sur deux Renards provenant d’un élevage hors enzootie canine. Par la suite, l’infestation spontanée du Renard par *L. infantum* s’est retrouvée dans plusieurs pays d’Europe méridionale. Dès lors, dans l’aire de *L. infantum*, le cycle du parasite pouvait être considéré comme primitivement sylvatique (« cycle primaire » de Garnham), avec un Canidae sauvage comme réservoir. Secondairement, ce cycle s’était « anthropisé », par passage sur le Chien domestique (« cycle secondaire » de Garnham). On imagine volontiers une étape intermédiaire, comportant, à la fois, des hôtes sauvages et domestiques (« cycle primo-secondaire » de Garnham). Notons enfin qu’en raison de la prévalence faible dans les populations humaines et en dehors des possibilités d’infester expérimentalement le vecteur sur des patients atteints d’immunodéficience acquise compliquée de leishmaniose viscérale, l’Homme demeurait une « impasse parasitaire ». À son terme, l’étude avait pleinement rempli ses objectifs : le cycle zoonotique de *L. infantum*, enfin « bouclé », apparaissait comme un excellent modèle d’écologie parasitaire. Les concepts de base avaient été développés : celui de complexe pathogène, d’épidémiologie du paysage et de zonage phyto-écologique, de système cyclique compartimenté, de plurifactorialité et d’évolution spatio-temporelle des foyers (changements climatiques et socio-économiques). Au surplus, pour la première fois en parasitologie de terrain, la notion de « transdisciplinarité » avait démontré son efficacité [13]. Néanmoins, un élément fondamental manquait au tableau : celui de l’identification précise des agents pathogènes, les espèces de *Leishmania* étant bien différentes sur le plan nosologique et épidémiologique mais indistingables morphologiquement. Pour parfaire l’analyse « bio-géographique » des foyers de transmission, il fallait une identification rigoureuse et indubitable des populations de parasites circulants. Morphologiquement univoques, ceux-ci ne pouvaient pas non plus être distingués en culture, comme c’était le cas pour les bactéries par exemple ; les tentatives basées sur une inoculation à l’animal n’avaient pas conduit beaucoup plus loin, pas plus que celles qui utilisaient l’immunologie (anticorps monoclonaux). Un outil nouveau a permis d’établir la systématique de l’énorme complexe pathogène leishmanien : l’identification isoenzymatique. Dès la publication de sa mise au point par le Pr Michael Chance (Liverpool), Jean-Antoine Rioux a importé la technique du typage par les isoenzymes, méthode la plus robuste pour l’époque, qu’il va parfaire, avec l’aide de Francine Pratlong, jusqu’à faire de Montpellier la référence mondiale dans le domaine [1]. Les « zymodèmes » de Montpellier (MON-...) restent ainsi aujourd’hui la référence pour l’O.M.S. Ainsi, pendant une décennie, utilisant de manière inédite et rigoureuse l’outil des isoenzymes, son équipe a approfondi la taxonomie et la systématique des *Leishmania*, aboutissant en 1990 à une révision complète de celle-ci, qui encore aujourd’hui, avec plus de 500 citations, reste l’article de référence en la matière [9]. Très tôt a également été créé un « Centre international de cryoconservation, d’identification enzymatique et d’étude taxonomique des *Leishmania* » qui, sans entrer dans les détails, se composait de quatre unités interconnectées : l’unité de cultures, la banque de « cryostabilats », le service d’identification et la structure de gestion et d’aide à la classification. Aboutissement du processus, la « banque de souches », reconnue officiellement par l’Organisation mondiale de la Santé et soutenue par le Centre national de la Recherche scientifique, abritait au départ à la retraite de Jean-Antoine Rioux près de 2500 souches de *Leishmania*, rigoureusement identifiées et désignées par des codes officiels (LEM et OMS). Ces deux activités d’expertise serviront de fondation à la création en 1998 du Centre National de Référence des Leishmanioses, par son successeur, Jean-Pierre Dedet. La taxonomie et la systématique sont restées par la suite ses disciplines de prédilection. Longtemps après sa retraite, et jusqu’à ses derniers jours, Jean-Antoine Rioux a approfondi sa pensée et continué de publier sur la systématique, l’épistémologie, l’histoire et la philosophie des sciences. Ouvert d’esprit et fortement intéressé par les avancées de la biologie moléculaire, il a jusqu’au bout espéré une systématique complète, réunissant les apports du phénotype apporté par les isoenzymes et des génotypes apportés par le séquençage de l’ADN. L’étude de deux foyers proches, la Corse et la Catalogne, confirma la validité des concepts mis en œuvre dans les Cévennes. Le premier était connu pour abriter une leishmaniose canine enzootique et ses conséquences humaines, sous la forme de cas sporadiques de leishmaniose viscérale. L’infestation canine fut systématiquement recherchée, sur environ 30 000 animaux, et les Leishmanies responsables cultivées et identifiées. Parallèlement, les Phlébotomes furent recherchés dans l’ensemble de l’île. Les résultats s’avérèrent très semblables à ceux qui avaient été trouvés sur le continent. Sans détailler ces résultats, soulignons qu’ils furent l’occasion de fructueux et fréquents échanges avec l’équipe italienne (Pr. Biocca, Pr. Coluzzi) qui travaillait dans la Sardaigne voisine. Par contre, en Pays catalan, le « complexe pathogène » leishmanien se distinguait de celui du Languedoc par la relative abondance de *P. perniciosus* par rapport à *P. ariasi*, et par la présence de *P. sergenti*, vecteur reconnu de *L. tropica*. Quelques années plus tard, des leishmanioses humaines strictement cutanées, dénommées « boutons d’Orient » autochtones, s’avéraient fréquentes dans le département des Pyrénées-Orientales. Bien plus, la taxonomie enzymatique, mise au point entre temps, rattachait ces lésions, non à *L. tropica*, comme nombre d’entre nous le supposaient, mais à *L. infantum*. Plus encore, outre la présence du classique zymodème MON-1, plusieurs autres zymodèmes étaient détectés, dont certains inconnus jusqu’alors : MON-29, MON-11 et MON-33, particulièrement liés aux formes cutanées. Plusieurs enquêtes de terrain, de part et d’autre de la frontière franco-espagnole, ont précisé les caractéristiques de ce foyer. Comme pour l’enquête Cévennes, la fréquence des leishmanioses canines a été établie. L’infestation canine a été confirmée par l’isolement de 27 souches de *Leishmania*, toutes rapportées au zymodème MON-1. L’analyse du volet Phlébotome s’est appuyée sur la technique des pièges adhésifs, selon la méthode des transects. 13.635 Phlébotomes ont été récoltés, appartenant aux genres *Phlebotomus* et *Sergentomyia*. L’impact humain a été déterminé à l’aide du test de Monténégro, réalisé sur 718 enfants. L’analyse statistique des résultats a montré une bonne corrélation entre les fréquences altitudinales des trois volets du tryptique. Ces fréquences présentaient un maximum entre 300 et 600 m. et deux minimums, l’un au-dessous de 150 m, l’autre au-dessus de 600 m. Sur le terrain, l’abondance maximale correspondait à la forêt mixte de *Quercus ilex* et *Q. pubescens*, étage considéré depuis lors, en Cévennes comme une « zone à risque ». Les dissections de *P. ariasi* et *P. perniciosus* réalisées dans les environs de Céret ont permis d’isoler et d’identifier deux des zymodèmes déjà observés chez l’Homme dans les leishmanioses cutanées autochtones : *L. infantum* MON-1 et MON-29. Les mêmes résultats ont été obtenus lors d’une enquête comparable en Catalogne espagnole. Par contre, la recherche de *Leishmania* chez des micromammifères (*Rattus rattus*, espèce incriminée dans le cycle de *L. infantum* en Italie) est demeurée négative. La transposition réussie des concepts dans un foyer proche mais différent dans sa structure a confirmé leur validité. Leur universalité l’a ensuite été par leur application à un grand nombre d’autres foyers et par leur succès dans la communauté internationale des « leishmaniaques ». Inlassablement remis en cause et affinés par leur auteur, ils sont restés jusqu’au bout la trame des travaux qu’il n’a cessé de publier, jusqu’à ses derniers mois [4,15]. De la même manière, le Centre de cryoconservation, d’identification enzymatique et d’étude taxonomique des *Leishmania*, élément fondateur du Centre National de Référence des Leishmanioses actuel, a été l’objet d’études fructueuses pendant toute la carrière de son concepteur, et a été transmis à son successeur à sa retraite. Animé par une équipe de scientifiques et de techniciens de tout premier ordre, sans cesse perfectionné, il a été l’outil indispensable des travaux menés par ce qui était devenu « l’École de Montpellier » et par tous ceux qui ont collaboré avec elle, Français et étrangers. Nombreuses ont été les recherches, menées en Europe (France, Espagne, Italie, Chypre), en Afrique du Nord (Maroc, Algérie, Tunisie), au Proche et au Moyen Orient (Égypte, Yémen, Syrie, Iraq, Oman), en Afrique sub-saharienne (Tchad, Sénégal) et en Amérique latine (Colombie, Équateur) confirmant la valeur de ce concept. Au fil des enquêtes, de nombreuses applications s’en sont dégagées, telles celles de risque temporo-spatial, de cartographie phytogéographique, d’anthropisation (paupérisation, désertification), de « lutte raisonnée » et, récemment, de relation avec les bioclimats. Il faut aussi souligner la qualité des relations établies avec les innombrables équipes des pays dans lesquels se sont déroulées ces recherches : relations scientifiques bien entendu, mais aussi relations amicales, l’enthousiasme et le savoir encyclopédique de Jean-Antoine Rioux forçant l’admiration, et son charisme séduisant ceux qui participaient aux recherches. Ses missions sur le terrain, en particulier en Cévennes, attiraient aussi nombre de scientifiques étrangers, sur lesquel(le)s elles ont laissé une empreinte indélébile. Deux Colloques internationaux, tenus à Montpellier en 1974 (sous l’égide de l’Inserm) et en 1984 (sous l’égide du CNRS et de l’OMS), sont venus couronner ces deux décennies de travaux en éco-épidémiologie et en taxonomie des leishmanioses. Ils ont réuni plus de 100 participants venus du monde entier (Angleterre, États-Unis, Belgique, Allemagne, Italie, Espagne, Portugal, Iran, Israël, URSS, Brésil, Venezuela, Colombie, Maroc, Algérie, Tunisie…), parmi lesquels des sommités dans le domaine que ce soit en épidémiologie ou en typage biochimique ou moléculaire. Les deux ouvrages issus de ces manifestations constituent encore aujourd’hui des références incontournables dans le domaine [5,8].

Il n’est pas envisageable de détailler ici toutes ces recherches. L’une d’elles, au Maroc, a été retenue pour les illustrer, parce qu’elle a été rendue possible par une coopération exemplaire, parce qu’elle s’est déroulée sur environ trente ans, et parce qu’elle a permis la compréhension d’une situation très complexe et débouché sur des stratégies de lutte et de prévention [11]. Dans ce pays, comme dans la plupart des pays circumméditerranéens, les leishmanioses ont toujours représenté un important problème de Santé publique. Qu’elles soient viscérales ou cutanées, zoonotiques ou anthroponotique, ces affections sont présentes du Nord au Sud, depuis les cédraies du Rif jusqu’aux palmeraies de l’Anti-Atlas, avec pour agents pathogènes *L. infantum*, *L. major* et *L. tropica*. Les recherches ont été menées par le Ministère de la santé du Maroc (Direction de l’Épidémiologie) et les laboratoires de Montpellier (Université Montpellier1/CNRS, Écologie parasitaire), avec des participants de Reims (Faculté de Pharmacie, Parasitologie,) Barcelone (Faculté de Pharmacie, Parasitologie,) Medellin (Faculté des Sciences, Colombie), et Trujillo (Faculté de Médecine, Venezuela). Plusieurs organismes extra-universitaires ont contribué au financement de l’opération : Ministères de la Coopération français, marocain et espagnol, Ministère des Affaires étrangères, OMS, UE, CNRS, INSERM. L’enquête a débuté en 1970, par l’étude taxonomique et chorologique des Phlébotomes. 19 espèces ont été inventoriées, dont plusieurs nouvelles pour le Maroc et d’autres nouvelles pour la science. Les densités de chaque espèce, exprimées en classes de fréquences par station, ont été traitées par l’analyse factorielle des correspondances. Grâce aux cartes thématiques, chaque station a pu être rapportée à son étage phytoécologique et sa zone bioclimatique. Chacune des espèces considérées comme vectrices de leishmanies a été située de façon précise. Une nouvelle fois, la prééminence du facteur climat dans la répartition géographique et l’abondance des vecteurs et, par voie de conséquence, dans la distribution et la force d’infection des foyers est apparue de manière indiscutable. Mais au-delà de l’enquête entomologique, un long chemin restait à parcourir pour parvenir à la connaissance structurale et dynamique des différents foyers marocains. Il devait durer deux décennies. Aujourd’hui, la couverture des leishmanioses, tant viscérale que cutanées, est en grande partie réalisée. La plupart des cycles épidémiologiques ont été établis grâce à l’identification enzymatique des *Leishmania* chez les vecteurs, les réservoirs et l’Homme. Pour chaque foyer, les risques potentiels peuvent être évalués, y compris ceux liés à d’éventuels changements climatiques. ln fine, plusieurs sites pilotes ont fait l’objet d’opérations de « lutte raisonnée ». Conduites par le Ministère de la Santé publique du Maroc, ces opérations ont été valorisées grâce à la mise en place d’un observatoire de veille épidémiologique. Chacune des 3 espèces leishmaniennes a fait l’objet d’études approfondies. Le premier foyer marocain de leishmaniose cutanée humaine à *L. major* fut signalé à la fin des années 1970, au sud de l’Anti-Atlas, dans la circonscription de Tata. L’identification enzymatique a permis de rapporter les 51 souches isolées chez l’Homme à *L. major* MON-25, zymodème observé sur l’ensemble du Maghreb, du Maroc à la Lybie. Les recherches ont porté sur la mise en évidence d’un réservoir de la famille des Gerbillidae et d’un vecteur du sous-genre *Phlebotomus*. Sur un total de 484 Rongeurs, seul *Meriones shawi grandis* était infesté par *L. major* MON-25 (12 souches identifiées, prévalence globale 14 %). L’affection, strictement cutanée, pouvait persister plusieurs années sans affecter le Mérion atteint. Toutefois, une intense diffusion parasitaire survenait en fin d’évolution. Cette longue tolérance cutanée, suivie par une courte phase de dissémination, conférait à *M. shawi* la qualité de « vrai réservoir » de *L. major* [7]. Son biotope optimal était constitué par des fosses circulaires creusées par l’homme à proximité des habitations. Initialement utilisées pour la fabrication des briques d’argile crue, ces excavations servaient par la suite de dépotoirs et de latrines. Devenu détritivore et stercoraire, le Rongeur se comportait alors comme un dangereux commensal. Parallèlement, l’infestation vectorielle était mise en évidence à 70 km à l’est de Tata. Mille six cent soixante-treize *Phlebotomus* spp. femelles étaient disséqués : seul *P. papatasi* était trouvé porteur de *L. major*. Il s’agissait du même zymodème MON-25. Pour contrôler l’enzootie, il était dès lors proposé la suppression physique des biotopes de *M. shawi*, des campagnes rodenticides, le suivi régulier de la densité des populations de Rongeurs. Chez l’homme, en parallèle, il était recommandé un dépistage de masse et un traitement pour réduire la durée d’évolution des lésions. Au Maroc méridional, l’aire de répartition d’une autre espèce du parasite, *L. tropica,* couvre 40.000 km^2^. Sur le versant nord du Haut-Atlas, la « zone à risque » s’étend d’est en ouest, en une frange continue, entre 500 et 1.000 m. d’altitude. Cette zone correspond à l’étage semi-aride à Tuya de Barbarie. Dans la plupart des sites inventoriés, la LCH s’exprime selon le « mode rural », c’est à dire hypo­endémique, dispersé et instable (incidence moyenne 5 %). L’importance du polymorphisme enzymatique de *L. tropica* dans le foyer du Haut-Atlas (MON- 102, MON-107, MON-112, MON-113, MON- 122, MON-123) et la répartition des zymodèmes dans trois sous-groupes phénétiques distincts, évoquaient des apports successifs d’origine orientale, plutôt qu’une différenciation *in situ*, à partir d’un ancêtre marocain. Dès les premières enquêtes, les formes infiltratives diffuses et pseudo-lépromateuses, déjà observées au Yémen et en Syrie chez les personnes âgées, étaient retrouvées au Maroc. Ces lésions, d’évolution prolongée, riches en amastigotes, constituaient une source importante de parasites, à la disposition du vecteur. Dans ces cas précis, le cycle de *L. tropica* paraissait bien de nature anthroponotique. Dans les foyers du Haut Atlas, comme au nord de la Syrie, le Chien domestique était porteur de lésions strictement cutanées à *L. tropica*. Toutefois, l’animal ne semblait pas constituer un « vrai » réservoir, malgré son taux d’infestation élevé (12 %) et sa contamination par les zymodèmes MON-102 et MON-11, également observés chez l’Homme. Chez le Chien, les lésions cutanées étaient en effet discrètes et fugaces et aucun cas de généralisation n’était observé. Notons que dans les foyers marocains dus à *L. tropica*, le Chien était souvent parasité par *L. infantum* MON-1. *P. sergenti*, vecteur reconnu de *L. tropica*, était abondant dans tous les foyers marocains. Les souches isolées se répartissent entre les zymodèmes MON-102, MON-107, MON-122 et MON-123, ces deux derniers jamais observés chez l’Homme ou le Chien. Comme pour *L. major* avec *P. papatasi*, la période à risque pour *L. tropica* avec *P. sergenti* se situait en fin de saison chaude. Les recommandations pour la lutte contre la LCH à *L. tropica* ne comportaient pas de lutte insecticide en raison du caractère hypo-endémique de la maladie, de sa distribution « dispersée », des difficultés d’accès aux douars contaminés et du coût des opérations. Elles devaient reposer sur un dépistage actif des cas et le traitement consécutif des lésions. Cette stratégie, éprouvée dans le site de Tanant a pu être généralisée à l’ensemble des foyers. Au Maroc, comme dans les autres Pays du Maghreb, les leishmanioses viscérales, tant humaines que canines, sont connues depuis plusieurs décennies. Par contre la distribution et l’importance des foyers zoonotiques, l’identification des « vrais » vecteur ainsi que l’existence de formes cutanées humaines demeuraient des problèmes d’actualité. La plupart provenaient de zones de moyenne altitude, du Rif et des Atlas. Le parasite appartenait à *L infantum* MON-1. Toutefois, la leishmaniose à *L infantum* ne se résumait pas à la seule forme viscérale. En 1990, une centaine de cas de leishmaniose cutanée humaine était dépistée dans la circonscription de Taghjicht. Le parasite était rapporté à *L. infantum* MON-24, zymodème habituellement responsable de cas sporadiques. L’enquête entomologique a montré que *P. longicuspis* s. l. était le seul *Larroussius* présent dans le site infesté de promastigotes (souche non isolée). *L. infantum* s’est finalement avérée présente sur la quasi-totalité du territoire marocain. Cette vaste distribution s’expliquait par la présence de trois vecteurs, *P. ariasi*, *P. perniciosus*, *P. longicuspis*, dont les préférences bioclimatiques et par conséquent les répartitions géographiques, différaient. *P. perniciosus*, à tendance semi-aride/subhumide, était surtout présent dans le Rif et le Moyen-Atlas. *P. ariasi* à tendance humide/subhumide l’accompagnait dans le Rif, tout en s’étendant sur le versant septentrional du Haut­Atlas. *P. longicuspis* et ses éventuels binômes jumeaux, à tendance aride/péri-aride cohabitait avec les espèces précédentes, mais s’observait seul aux étages aride, chaud et saharien. En d’autres termes, au Maroc, la succession Nord-Sud des vecteurs et leur association dans les interfaces bioclimatiques assuraient à *L. infantum* une couverture géographique quasi continue.

### Conclusions

Les recherches présentées dans ces quelques pages sont loin d’être les seules qu’a effectuées le travailleur infatigable qu’était le Pr. Jean-Antoine Rioux. Son épreuve de titres et travaux fait état de plus de 500 publications, auxquelles il faut ajouter les ouvrages, les thèses encadrées… Pour reprendre les termes de l’un d’entre nous (Patrick Bastien, son successeur actuel dans le laboratoire de Parasitologie de Montpellier) : « Monsieur Rioux était un de ces hommes qu’on ne croise que trop rarement et qu’on ne peut oublier après l’avoir connu ». Président de la Société française de Parasitologie de 1982 à 1999, il l’a amenée au rang qu’elle occupe actuellement, notamment en la dotant de statuts modernes. Il a su fédérer ses membres pour organiser à Paris, en 1990, un congrès international (ICOPA7) qui a réuni plus de 2500 participants. Terminons en ajoutant que cet enseignant brillant, maniant avec dextérité une langue irréprochable, était aussi un homme cultivé, et qu’il n’a jamais cessé d’être, pour celles et ceux qui l’ont accompagné au long de ces années, un ami également inoubliable. Jean-Antoine Rioux était Chevalier de l’Ordre National de la Légion d’Honneur, Officier de l’Ordre National du Mérite, Officier de l’Ordre des Palmes Académiques, Chevalier de l’Ordre du Mérite Agricole. Il était également membre de plusieurs académies : Académie Royale de Médecine de Belgique, Académie Royale des Sciences d’Outre-Mer (Bruxelles), Académie des Sciences et Lettres de Montpellier (Président en 1996) et Académie du Languedoc (Toulouse). Il était membre de nombreuses Sociétés savantes : Société des Naturalistes parisiens, Société Botanique de France Association des Anciens Élèves de l’Institut Pasteur, Société Française de Mycologie Médicale, Société Française de Systématique (Membre d’honneur), Société d’Horticulture et d’Histoire Naturelle de l’Hérault, Société de Pathologie Exotique, Société Internationale de Mycologie Humaine et Animale, Société Française de Protozoologie, membre fondateur de la Société de Protection de la Nature du Languedoc-Roussillon (Président en 1972), membre fondateur de la Société Française d’Écologie (Président de 1977 à 1981), membre fondateur de la Société Française de Parasitologie (Président de 1982 à 1999 et Président d’honneur depuis 2003).
